# Personal

**Published:** 1906-03

**Authors:** 


### PERSONAL.

DR. H. E. HAYD, of 493 Delaware Avenue, Buffalo, entertained a company of men at dinner February 27, 1906, at his charming home, in honor of Dr. Hunter Robb, of Cleveland, who read a paper the same evening before the Buffalo Academy of Medicine.

---

DR. JOHN H. PRYOR, of Sarnac Lake, visited Buffalo recently, where he received a cordial greeting from many of his old friends and colleagues. He was the guest of honor at a dinner given at the Saturn Club, Saturday evening, March 3, 1906, at which covers were laid for sixteen.

---

DR. CHARLES A. L. REED, of Cincinnati, has been elected president of the Smoke Abatement League of that city. Dr. Reed's well-known interest and activity in promoting sanitary reforms, will have ample opportunity for exhibition during the current year, in the exercise of the functions of this important office.

---

DR. R. E. FORT, of Nashville, has been appointed surgeon in chief of the Nashville division of the Southern Railway, and Surgeon of the Eastern Division of the Illinois Central Railroad.

---

DR. HENRY BEATES, JR., of Philadelphia, has been reappointed a member of the Pennsylvania State Board of Medical Examiners. Dr. Beates has served for some years as president of the board, and is also president of the National Confederation of State Examiners.

---

DR. HERBERT NORTHRUP SQUIER, a graduate of the University of Buffalo, 1904, who has been an interne at the Buffalo General

Hospital for eight months, will become house physician at Saint Luke's Hospital, Utica, N. Y., on the retirement of Dr. William H. Boothe, who enters upon private practice very soon.

---

Dr. Bernard Bartow, of Buffalo, has been elected president, and Dr. Irving M. Snow, one of the vice-presidents of the Park Club.

---

Hon. St. Clair McKelway, vice-chancellor of the Board of Regents of the State of New York, was elected, February 13, 1906, by the Houses of the Legislature, meeting separately, to succeed himself as Regent for the full term of eleven years, beginning April 1. Dr. McKelway is one of the most active regents and his election will prove gratifying to everyone interested in the educational affairs of the state. We publish elsewhere in this issue his admirable address, delivered at the centenary of the Medical Society of the State of New York.

---

Dr. William Mabon, for sometime past chairman of the State Commission in Lunacy, was recently appointed superintendent of the Manhattan State Hospital, Ward's Island, N. Y. Dr. Mabon is recognised as one of the leading alienists in this country. country.

